# Risk factors for onset or progression of epiretinal membrane after cataract surgery

**DOI:** 10.1038/s41598-021-94352-9

**Published:** 2021-07-20

**Authors:** Soonil Kwon, Boyun Kim, Sohee Jeon

**Affiliations:** 1 Department of Ophthalmology, Hallym University Sacred Heart Hospital, Anyang, Republic of Korea; 2 Keye Eye Center, 326 Teheran-ro, Gangnam-gu, Seoul Republic of Korea

**Keywords:** Eye diseases, Biomarkers

## Abstract

While the precise diagnosis of early stage epiretinal membrane (ERM) at the time of cataract surgery and evaluation of risk factors for development or progression of ERM after cataract surgery is increasingly important, there is only limited information. In the present study, we evaluated the risk factors for onset or progression of ERM on spectral domain optical coherence tomography (SD-OCT) after cataract surgery. The univariate analysis showed that eyes with partial posterior vitreous detachment (PVD; *p* < 0.001), hyper-reflective foci (HF) on the inner retinal surface (*p* < 0.001), vitreoschisis (*p* = 0.014), and discrete margin of different retinal reflectivity (DMDRR; *p* = 0.007) on ultra-widefield fundus photography (UWF-FP) had significant risk for the onset or progression of ERM after cataract surgery. The multivariate analysis showed that partial PVD (HR, 3.743; 95% confidence interval [CI], 1.956–7.162; *p* < 0.001), HF (HR, 2.330; 95% CI, 1.281–4.239; *p* = 0.006), and DMDRR on UWF-FP (HR, 3.392; 95% CI, 1.522–7.558; *p* = 0.003) were the independent risk factors for the onset or progression of ERM after cataract surgery after adjustment for other confounding factors. Our study shows that the onset or progression of ERM after cataract surgery depends on an abnormal vitreoretinal interface (VRI) represented by partial PVD, HF on SD-OCT, and DMDRR on UWF-FP, not on age, axial length, or presence of ERM at the time of surgery. A meticulous funduscopic evaluation of the VRI would help to predict the ERM risk before cataract surgery.

## Introduction

The causal relationship between cataract surgery and epiretinal membrane (ERM) has been discussed extensively without definite conclusion^1–3^. A major hurdle in the evaluation of this relationship has been the poor diagnostic agreement in the detection of ERM by preoperative and postoperative fundus photography due to the poor penetration of light through the preoperative lens^4,5^. The diagnosis of ERM is becoming more objective as spectral domain optical coherence tomography (SD-OCT) becomes more widely used^6,7^. SD-OCT has strength for monitoring ERM after cataract surgery because the light can penetrate the media opacity even in dense cataracts^8^. In addition, SD-OCT enables detailed observation of the vitreomacular interface (VMI), with differentiation of vitreomacular adhesion (VMA) and vitreomacular traction (VMT) from posterior vitreous detachment (PVD), which is crucial for ERM formation^9,10^. Furthermore, a comparative study using SD-OCT and ultra-widefield fundus photography (UWF-FP) can provide additional information on the vitreoretinal interface (VRI) that is related to the ERM^11^.

With the growing incidence of multifocal intraocular lens (MIOL) implantation, precise diagnosis of early stage ERM at the time of cataract surgery and evaluation of risk factors for development or progression of ERM after cataract surgery is increasingly important. Unlike cases of clinically significant ERM that distorts the outer retinal structure and eventually requires surgical removal, the clinical prognosis of clinically insignificant ERM that does not affect vision at the time of cataract surgery is open to discussion. We found that the visual quality after MIOL is not favorable in eyes with mild ERM, even when the ERM did not involve the fovea^12^. Moreover, the age of patients at cataract surgery with MIOL implantation is younger^13^, which puts them at greater lifetime risk of ERM development or progression after surgery.

At this point, there is consensus on aging, PVD, and cataract surgery as the risk factors for the development of ERM in eyes without underlying retinal disease^14,15^. Theoretically, VMT during PVD can cause ERM, especially when the PVD process is not coupled with proper liquefaction of vitreous. The role of cataract surgery in ERM formation would also be related to iatrogenic PVD associated with anteroposterior movement of the vitreous. However, there is only limited information about the relationship between these pre- and intraoperative risk factors and ERM. Therefore, in the present study, we evaluated the risk factors for the development or progression of ERM after cataract surgery based on various ocular parameters that include meticulous evaluation of retinal images from SD-OCT and UWF-FP in a relatively large cohort.

## Results

**Figure 1:**
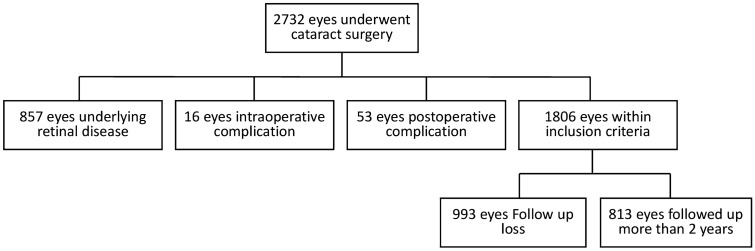
Flowchart illustrating the distribution of patients.

Of the 1806 eyes within the inclusion criteria that underwent cataract surgery during the study period (total 2732 eyes), 813 eyes had more than 2 years of follow-up (Fig. 1). Table 1 shows the baseline characteristics of eyes with and without ERM progression after cataract surgery. The mean age of enrolled patients was 59.28 ± 5.96 years and 262 out of 813 patients were men. Among 813 eyes, there were 59 eyes (7.3%) that showed onset or progression of ERM during a follow-up period of 27.45 months. There was no difference in age at cataract surgery between groups (59.32 ± 6.01 years for no onset or progression group vs 58.64 ± 5.32 for onset or progression group, *p* = 0.397), and there also were no differences in sex, follow-up period, UDVA, CDVA, phacoemulsification time, or CDE (*p* = 0.236; *p* = 0.341; *p* = 0.991; *p* = 0.294; *p* = 0.146; and *p* = 0.883, respectively; Table 1). There was no difference in ocular biometry, including Km, Ka, TCIA, AL, ACD, and LT between groups (*p* = 0.391; *p* = 0.077; *p* = 0.810; *p* = 0.842; *p* = 0.154; and *p* = 0.802, respectively; Table 1).

**Table 1:** Baseline characteristics of subjects in the present study.

	Total (n = 813)	No onset or progression of ERM (n = 754)	Onset or progression of ERM (n = 59)	*p* value
Age, years	59.28 ± 5.96	59.32 ± 6.01	58.64 ± 5.32	0.397*
Sex, male (%)	262 (32.2)	246 (32.6)	16 (27.1)	0.236^†^
Follow-up periods, months	27.45 ± 3.99	27.41 ± 3.97	27.93 ± 4.19	0.341*
Phacoemulsification time, seconds	11.01 ± 10.63	11.21 ± 10.79	9.02 ± 8.72	0.146*
CDE, %-seconds	1.03 ± 1.45	1.03 ± 1.14	1.00 ± 1.54	0.883*
UDVA, LogMAR	0.34 ± 0.32	0.34 ± 0.32	0.34 ± 0.31	0.991*
CDVA, LogMAR	0.03 ± 0.11	0.03 ± 0.11	0.02 ± 0.05	0.294*
Km, D	43.63 ± 1.52	43.61 ± 1.51	43.78 ± 1.63	0.391*
Ka, D	0.82 ± 0.65	0.80 ± 0.64	0.96 ± 0.73	0.077*
TCIA, D	0.16 ± 0.13	0.16 ± 0.13	0.17 ± 0.08	0.810*
AL, mm	23.83 ± 1.33	23.82 ± 1.35	23.86 ± 1.14	0.842*
ACD, mm	2.98 ± 0.74	2.97 ± 0.76	3.11 ± 0.32	0.154*
LT, mm	4.45 ± 0.33	4.45 ± 0.33	4.46 ± 0.27	0.802*

*ACD* anterior chamber depth, *AL* axial length, *CDE* cumulative dissipated energy, *CDVA* corrected distant visual acuity, *Ka* keratometric astigmatism, *Km* mean keratometric value, *LT* lens thickness, *TCIA* total corneal irregular astigmatism, *UDVA* uncorrected distance visual acuity.

Data are mean ± standard deviation unless otherwise indicated. Student’s t-test* or Chi-square test^†^ was used.

Table 2 shows ocular parameters from SD-OCT and UWF-FP in each group. There was no significant difference in CST at baseline (264.36 ± 23.28 µm for no onset or progression group vs 268.10 ± 21.19 µm for onset or progression group, *p* = 0.330). Also, the presence of ERM at the time of surgery had no relationship with the onset or progression of ERM during the follow-up period (*p* = 0.324). There were significant differences in VMI status at baseline, change in PVD status, HF on inner retinal surface, vitreoschisis, and DMDRR on UWF-FP between groups (*p* < 0.001; *p* < 0.001; *p* < 0.001; *p* = 0.023; and *p* = 0.011, respectively). No difference was detected in presence of epiretinal mass and retinal breaks between groups (*p* = 0.522 and *p* = 0.252).

**Table 2:** Ocular parameters from SD-OCT and UWF-FP in the present study.

	Total (n = 813)	No onset or progression of ERM (n = 754)	Onset or progression of ERM (n = 59)	*p* value
CST, µm	264.60 ± 23.15	264.36 ± 23.28	268.10 ± 21.19	0.330*
ERM at baseline (%)	150 (18.5)	141 (18.7)	9 (15.2)	0.324^†^
**VMI status**				< 0.001^†^
No PVD (%)	210 (25.8)	203 (26.9)	7 (11.8)	
Partial PVD (%)	313 (38.5)	270 (35.8)	43 (72.9)	
PVD (%)	290 (35.7)	281 (37.3)	9 (15.3)	
Change in PVD status (%)	160 (19.7)	119 (15.8)	41 (69.5)	< 0.001^†^
Hyper-reflective foci on ILM (%)	65 (8.0)	48 (6.4)	17 (28.8)	< 0.001^†^
Vitreoschisis (%)	66 (8.1)	53 (7.0)	13 (22.0)	0.023^†^
Epiretinal mass (%)	35 (4.3)	33 (4.4)	2 (3.4)	0.522^†^
DMDRR (%)	72 (8.9)	61 (8.1)	11 (18.6)	0.011^†^
Retinal breaks (%)	18 (2.2)	18 (2.4)	0 (0.0)	0.252^†^

*CST* central subfoveal thickness, *DMDRR* discrete margin of different retinal reflectivity, *ERM* epiretinal membrane, *ILM* internal limiting membrane, *PSD* pachychoroid spectrum disease, *PVD* posterior vitreous detachment, *VMI* vitreomacular interface.

Data are mean ± standard deviation unless otherwise indicated. Student’s t-test* or Chi-square test^†^ was used.

**Figure 2:**
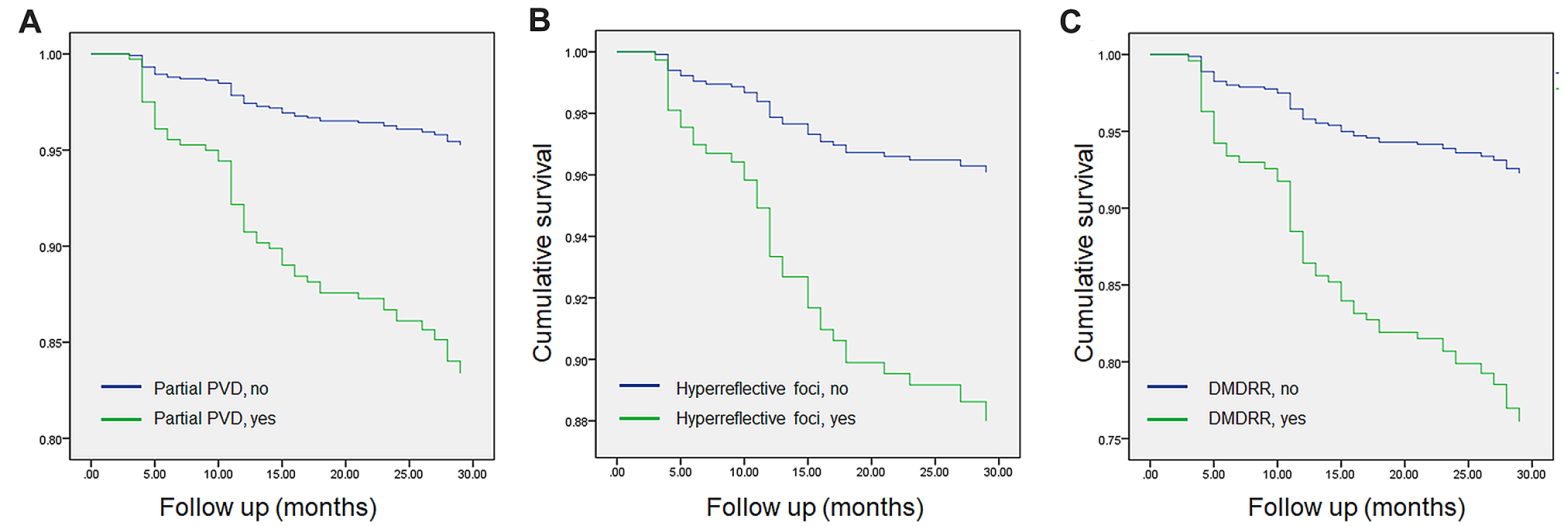
Cox proportional hazard ratio analysis. Cumulative hazards according to (**A**) presence of partial posterior vitreous detachment (PVD); (**B**) hyperreflective foci on inner retinal surface; and (**C**) discrete margin of different retinal reflectivity (DMDRR) in the mid- to far-peripheral retina on ultra-widefield fundus photography.

A Cox proportional hazard analysis was performed to evaluate the risk factors after cataract surgery for the onset of ERM or the progression of ERM detected at the time of cataract surgery (Table 3). The univariate analysis showed that eyes with partial PVD at the macula (HR, 4.481; 95% CI, 2.524–14.929; *p* < 0.001), HF on inner retinal surface (HR, 3.610; 95% CI, 2.032–6.414; *p* < 0.001), vitreoschisis (HR, 2.112; 95% CI, 1.163–3.837; *p* = 0.014), and DMDRR (HR, 2.478; 95% CI, 1.286–4.774; *p* = 0.007) had significant risk for the onset or progression of ERM after cataract surgery. The multivariate analysis showed that partial PVD at the macula (HR, 3.743; 95% CI, 1.956–7.162; *p* < 0.001; Fig. 2A), HF on inner retinal surface (HR, 2.330; 95% CI, 1.281–4.239; *p* = 0.006; Fig. 2B), and DMDRR on UWF-FP (HR, 3.392; 95% CI, 1.522–7.558; *p* = 0.003; Fig. 2C) were the independent risk factors for the onset or progression of ERM after cataract surgery after adjustment for other confounding factors including age and sex.

**Table 3:** Cox proportional hazard model for prediction of ERM onset or progression after cataract surgery.

	Univariate analysis			Multivariate analysis		
	HR	95% CI	*p* value	HR	95% CI	*p* value
Age	0.981	0.939–1.025	0.383			
Sex	1.286	0.724–2.283	0.391			
CST	1.006	0.994–1.019	0.310			
ERM at baseline	0.811	0.399–1.652	0.565			
Partial PVD at macula	4.481	2.524–7.956	< 0.001	3.743	1.956–7.162	< 0.001
Hyperreflective foci on ILM	3.610	2.032–6.414	< 0.001	2.330	1.281–4.239	0.006
Vitreoschisis	2.112	1.163–3.837	0.014			
Epiretinal mass	0.774	0.189–3.170	0.722			
DMDRR	2.478	1.286–4.774	0.007	3.392	1.522–7.558	0.003
Retinal breaks	0.048	0.000–85.079	0.426			

*CST* central subfoveal thickness, *DMDRR* discrete margin of different retinal reflectivity, *ERM* epiretinal membrane, *ILM* internal limiting membrane, *PSD* pachychoroid spectrum disease, *PVD* posterior vitreous detachment, *VMI* vitreomacular interface.

**Figure 3:**
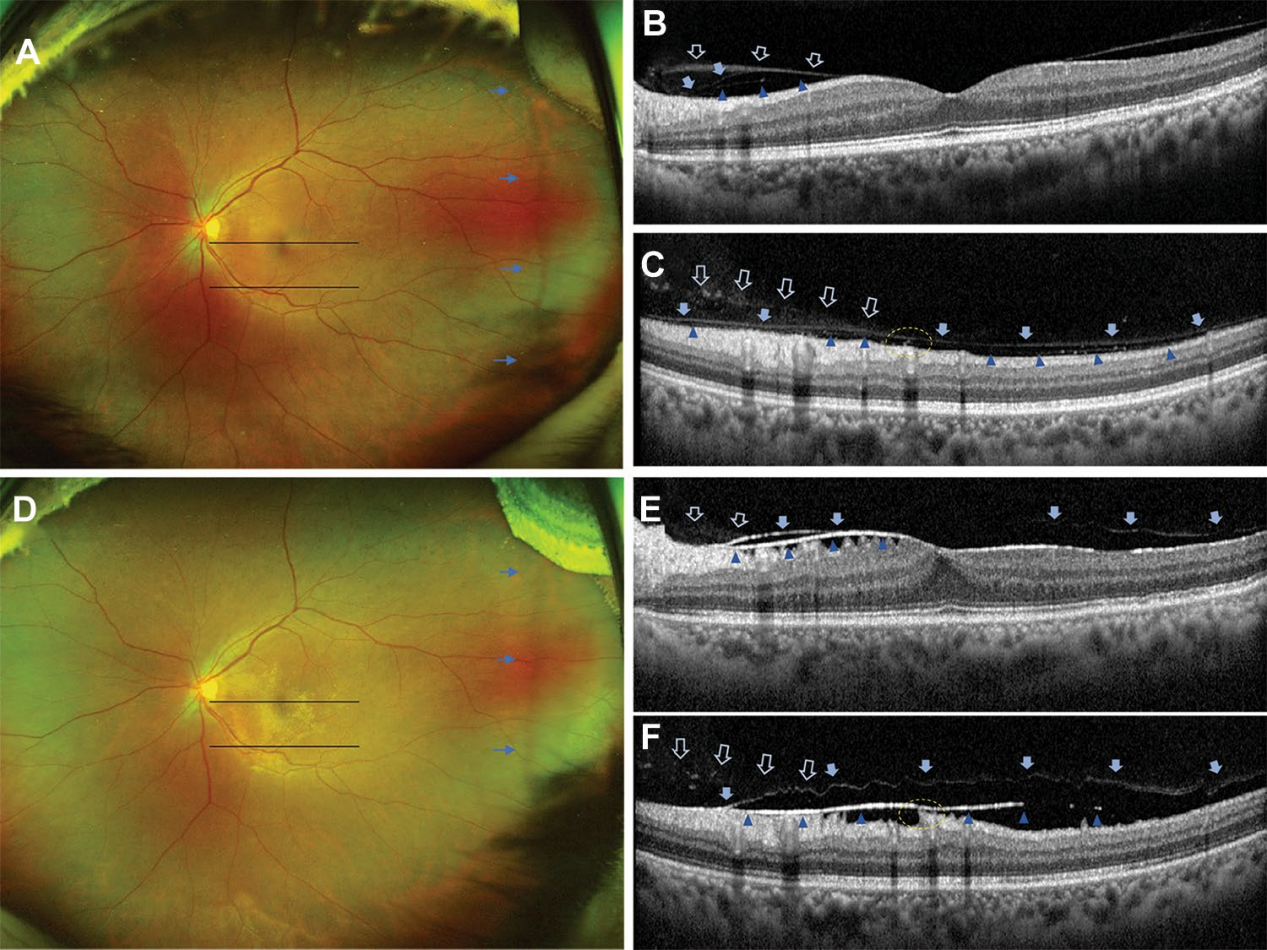
Representative images from a 61-year-old woman at preoperative (**A**–**C**) and 1-year follow-up (**D**–**F**). (**A**) A discrete margin of different retinal reflectivity (DMDRR; arrows) was presented on ultra-widefield fundus photography (UWF-FP). Two black lines indicate the position of figures (**B**) and (**C**). (**B**) Spectral domain optical coherence tomography (SD-OCT) image on fovea shows partial posterior vitreous detachment (PVD) with vitreoschisis. Each type of arrows and arrowheads indicates the separated vitreous cortex and posterior hyaloid membrane (PHM). (**C**) SD-OCT image at the inferior peri-macular area shows vitreoschisis and hyperreflective foci (HF) on the inner retinal surface (dotted circle). (**D**) One year later, the epiretinal membrane (ERM) was detected on the fovea. A DMDRR (arrows) was detected on the more posterior retina than 1 year ago. Two black lines indicate the position of figures (**E**) and (**F**). (**E**) SD-OCT image on fovea shows stage 3 ERM. Note that the nasal ERM shows two lines of hyperreflective lines that present identical locations of the separated vitreous cortex (arrows) and PHM (arrowheads) in figure (**B**). (**F**) SD-OCT image at the inferior peri-macular area shows ERM that locates at the identical location of PHM in figure C (arrowheads). Retinal tissues were dragged to the ERM at the location where the HF was detected (dotted circle) in figure (**C**).

Among 150 eyes with mild ERM before surgery, 9 (6.0%) showed progression of ERM. In 663 eyes without ERM at baseline, 50 (7.5%) showed new onset of ERM during the follow-up period. Since the eyes with and without ERM would have different risk factors, we further evaluated the risk factors for onset or progression of ERM after surgery in eyes with and without ERM preoperatively. Table 4 shows the Cox proportional hazard model for ERM onset after cataract surgery in eyes without ERM at baseline. The multivariate analysis showed that partial PVD at the macula (HR, 3.707; 95% CI, 1.483–9.528; *p* = 0.005), HF on the inner retinal surface (HR, 3.168; 95% CI, 1.577–6.366; *p* = 0.001), and DMDRR on UWF-FP (HR, 3.086; 95% CI, 1.077–8.841; *p* = 0.036) were the independent risk factors for new-onset ERM after cataract surgery in eyes without ERM at baseline after adjustment of other confounding factors including age and sex. On the other hand, in eyes with mild ERM at baseline, partial PVD at the macula (HR, 10.985; 95% CI, 0.994–121.379; *p* = 0.051) was the only risk factor for ERM progression after cataract surgery (Table 5). Figure 3 shows a representative case of ERM onset after cataract surgery in a 61-year old woman. The UWF-FP shows DMDRR at the temporal peripheral retina (Fig. 3A) and SD-OCT shows partial PVD with vitreoschisis and HF at the inner retinal surface (Fig. 3B, C) before cataract surgery. One year after uneventful cataract surgery, there is a new onset of ERM (Fig. 3D) with shrinkage of the posterior hyaloid membrane and underlying membranous changes was visible on SD-OCT (Fig. 3E, F).

**Table 4:** Cox Proportional Hazard Model for prediction of ERM onset after cataract surgery in eyes without ERM at baseline (n = 663).

	Univariate analysis			Multivariate analysis		
	HR	95% CI	*p* value	HR	95% CI	*p* value
Age	0.995	0.938–1.056	0.882			
Sex	0.972	0.429–2.205	0.946			
CST	1.017	1.004–1.030	0.012			
Partial PVD at macula	3.376	1.320–8.634	0.011	3.706	1.483–9.258	0.005
Hyperreflective foci on ILM	4.009	1.830–8.782	0.001	3.168	1.577–6.366	0.001
Vitreoschisis	0.948	0.454–2.021	0.890			
Epiretinal mass	0.464	0.062–3.496	0.456			
DMDRR	3.392	1.162–9.902	0.025	3.086	1.077–8.841	0.036
Retinal breaks	0.000	0.000–224,573	0.973			

*CST* central subfoveal thickness, *DMDRR* discrete margin of different retinal reflectivity, *ERM* epiretinal membrane, *ILM* internal limiting membrane, *PSD* pachychoroid spectrum disease, *PVD* posterior vitreous detachment, *VMI* vitreomacular interface.

**Table 5:** Cox proportional hazard model for prediction of ERM onset after cataract surgery in eyes with mild ERM at baseline (n = 150).

	Univariate analysis			Multivariate analysis		
	HR	95% CI	*p* value	HR	95% CI	*p* value
Age	0.841	0.699–1.011	0.065			
Sex	0.280	0.056–1.385	0.119			
CST	0.950	0.896–1.008	0.090			
Partial PVD at macula	4.322	1.159–16.115	0.029	10.985	0.994–121.379	0.051
Hyperreflective foci on ILM	4.421	0.526–37.144	0.171			
Vitreoschisis	0.047	0.000–3,211,447	0.740			
Epiretinal mass	1.341	0.167–10.759	0.783			
DMDRR	3.587	0.883–14.567	0.074			
Retinal breaks	0.047	0.000–826,658	0.720			

*CST* central subfoveal thickness, *DMDRR* discrete margin of different retinal reflectivity, *ERM* epiretinal membrane, *ILM* internal limiting membrane, *PSD* pachychoroid spectrum disease, *PVD* posterior vitreous detachment, *VMI* vitreomacular interface.

## Discussion

In the present study, we found that the risk of onset or progression of ERM after cataract surgery can be predicted by retinal characteristics using UWF-FP and SD-OCT. Eyes with partial PVD, HF on the inner retinal surface, or DMDRR at the time of cataract surgery had three times greater risk of onset or progression of ERM after cataract surgery. Among 313 eyes with partial PVD, 43 (13.7%) showed onset or progression of ERM during the postoperative period, while only 7 out of 210 eyes with no PVD (3.3%) and 9 out of 290 eyes with PVD at macular scan (3.1%) showed onset or progression of ERM during the postoperative period. We speculate that the HF on the inner retinal surface may represent a cluster of various cells and extracellular matrix, such as Muller glial cells and type IV collagen, which also proved to be a major source of ERM in a previous histologic study^16^. DMDRR is a relatively new finding that we have recently reported as a margin of ellipsoid zone disruption that may originate from vitreous traction^11^. All three of the above risk factors for the onset or progression of ERM may have a relationship with the vitreous traction resulting from focal strong vitreoretinal adhesion during the PVD process. Anteroposterior movement of the vitreous during the cataract surgery in eyes with focal strong vitreoretinal adhesion would result in an onset or progression of ERM. In addition, the presence of ERM at baseline did not increase the risk of ERM worsening (HR = 0.811 for onset or progression of ERM in the presence of ERM at baseline). When we analyzed the risks only in eyes with ERM at baseline, partial PVD was the sole risk factor for the onset or progression of ERM, suggesting that an abnormal VRI, and not ERM itself, is a risk factor for progression of ERM after cataract surgery.

Fong et al. reported a 3-year cumulative incidence of ERM detected by fundus photography of 11.2% after cataract surgery in an Australian cohort with mean age of 74.9 years^4^. In the present study, the 27-month cumulative incidence of onset or progression of ERM detected by SD-OCT was 7.3% after cataract surgery in a Korean cohort with a mean age of 59.3 years. We believe that the relatively young age and possibly the different ethnicity contributed to the lower incidence of ERM after cataract surgery even with the more sensitive diagnostic tool.

Increasing age has been significantly associated with idiopathic ERM (iERM) in previous population-based studies^2,17–23^, and female sex was associated with iERM after adjusting for age^19,24^. AL has also shown significant association with iERM after adjusting for age and sex^19,25,26^. In the present study, neither age or sex, nor AL were associated with increased risk for onset or progression of ERM after cataract surgery. It is possible that certain known risk factors for ERM, such as AL, would have a direct relationship with PVD status, rather than ERM. Further study in a large cohort is mandatory to address this issue.

We found that the onset or progression of ERM after cataract surgery varies depending on the characteristics of the VRI, including PVD status, HF on SD-OCT, and DMDRR on UWF-FP, but not on age, AL, or presence of ERM at the time of surgery. SD-OCT and UWF-FP are not the standard of care for cataract surgery worldwide. However, our findings suggest that a meticulous funduscopic evaluation of the VMI before cataract surgery would help to predict the ERM risk, especially when an MIOL is planned, as the onset or progression of ERM would impact the quality of vision. A prospective long-term study is warranted to confirm these findings.

## Methods

A retrospective chart review was performed for eyes with a history of phacoemulsification and IOL implantation from December 2017 to April 2019 at the KEYE Eye Center, Seoul, Korea. Data of patients who had been followed for at least 2 years after cataract surgery were reviewed. Exclusion criteria included eyes with retinal diseases including age-related macular degeneration, diabetic retinopathy, retinal vascular occlusions, or a previous history of any refractive or vitreoretinal surgery. Eyes with fovea-involving ERM were also excluded, while eyes with non-fovea-involving ERM were included. Eyes with intraoperative capsular damage or any kind of postoperative complication, such as cystoid macular edema or postoperative IOL exchange were also excluded. The Institutional Review Board (IRB)/Ethics Committee of KEYE Eye Center (IRB number 2020-0724-001) approved the study and waived the requirement for informed consent because of the retrospective nature of the study. The study protocol adhered to the tenets of the Declaration of Helsinki.

Routine preoperative ocular examinations included monocular uncorrected and corrected distance visual acuity (UDVA and CDVA), biomicroscopic examination, intraocular pressure (IOP) measurement, Pentacam Scheimpflug analysis (Oculus Inc., Berlin, Germany), optical biometry with a partial coherence interferometry device (IOL Master, Carl Zeiss Meditec, Jena, Germany), SD-OCT (Heidelberg Engineering, Heidelberg, Germany) and UWF-FP (Optos Optomap Panoramic 200A Imaging System; Optos plc, Dunfermline, Scotland). Biomicroscopic examination, indirect binocular ophthalmoscopy, and OCT scans were repeated at postoperative months 1, 2, 6, 12, 18, and 24.

The following parameters were examined for potential correlation with the development or progression of ERM: age; sex; phacoemulsification time; cumulative dissipated energy (CDE); CDVA and UDVA; IOP; mean keratometric value (Km), astigmatism (Ka), and total corneal irregular astigmatism (TCIA) from the Pentacam Scheimpflug system; axial length (AL), anterior chamber depth (ACD), and lens thickness (LT) from the IOL Master; presence of ERM; presence of PVD; changes in PVD status during the follow-up; central subfoveal thickness (CST); hyper-reflective foci (HF) on the internal limiting membrane (ILM); vitreoschisis and epiretinal mass indicating remnant vitreous cortex^10^; and a discrete margin of different retinal reflectivity (DMDRR)^11^ and retinal breaks on UWF-FP. All OCT and UWF-FP images were carefully evaluated by two investigators (S.K., B.K.), who were blinded to the patient groups and identities.

### Definition of ocular parameters from SD-OCT and UWF-FP

**Figure 4:**
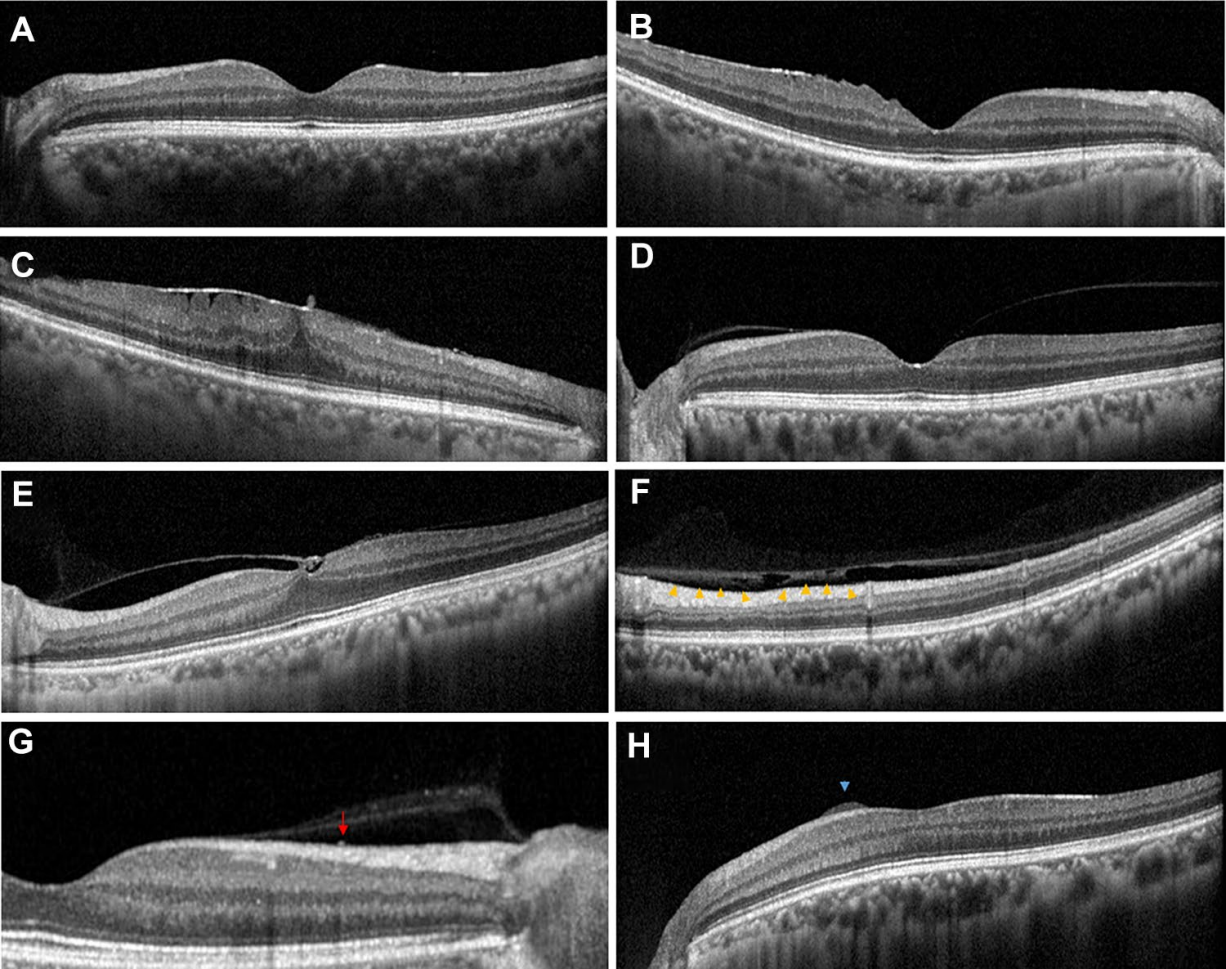
Representative images of risk factors detected by spectral domain optical coherence tomography (SD-OCT). (**A**) Stage 1 ERM; a continuous hyperreflectivity on the inner retinal surface on at least three consecutive scans of the macular volume. (**B**) Stage 2 ERM; stage 1 ERM with the presence of retinal folds but without associated foveal alteration. (**C**) Stage 3 ERM; stage 2 ERM with foveal involvement. (**D**) Vitreomacular adhesion. (**E**) Vitreomacular traction. (**F**) Vitreoschisis; a split in the posterior vitreous cortex (arrowheads). (**G**) Hyperreflective foci; discrete lesions protruding from the inner retinal surface. (**H**) Epiretinal mass; a homogenous collection of material with medium reflectivity at the inner retinal surface with a thickness greater than 20 µm.

**Figure 5:**
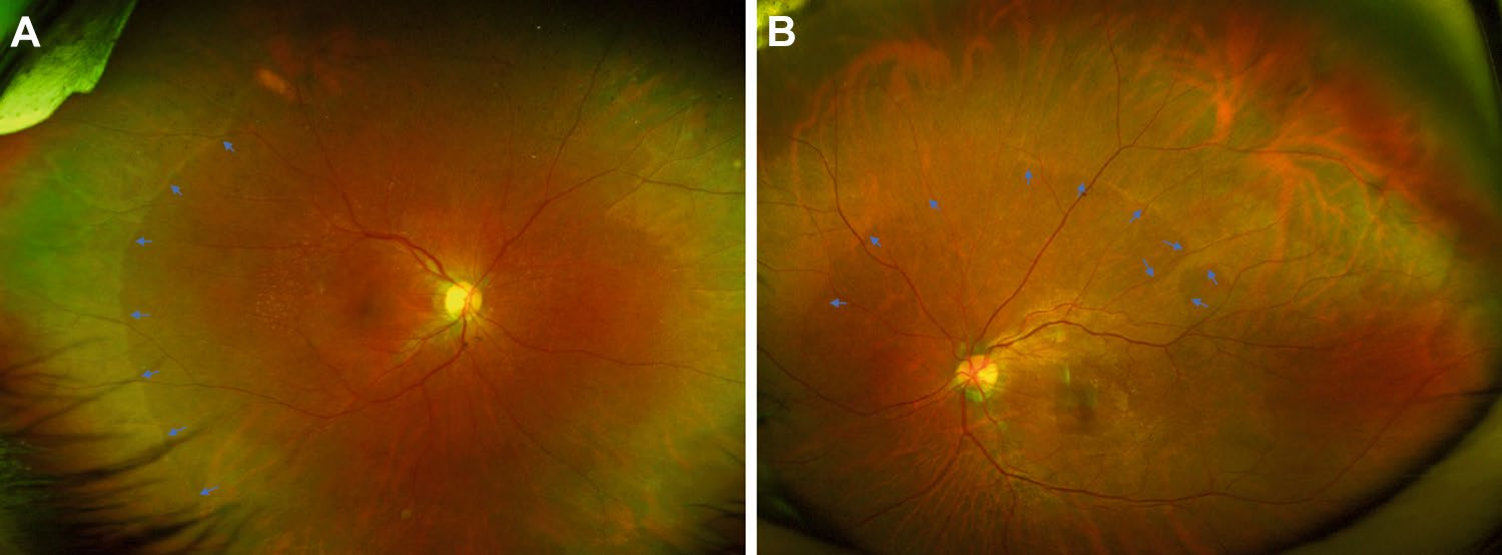
Representative images of eyes with discrete margin of different retinal reflectivity (DMDRR). (**A** and **B**) discrete margin of different retinal reflectivity in the mid- to far-peripheral retina on ultra-widefield fundus photography (arrows).

Figure 4 shows the representative images from enrolled patients. ERM in this study was defined as the presence of a continuous hyperreflective signal at the inner retinal surface on at least three consecutive sections of the macular cube to include early stage ERM^6^. Stage 1 ERM was diagnosed when a continuous hyperreflectivity on the inner retinal surface was observed on at least three consecutive scans of the macular volume (Fig. 4A). Stage 2 ERM corresponds to stage 1 ERM with the presence of retinal folds but without associated foveal alteration (Fig. 4B). Stage 3 ERM corresponds to stage 2 ERM with foveal involvement (Fig. 4C). Onset or progression of ERM was defined when there was new onset of ERM in eyes without ERM at baseline or progression of ERM stage in eyes with ERM at baseline. The vitreomacular interface (VMI) at baseline was classified as previously defined by the International Vitreomacular Traction Study (IVTS) Group^27^ with slight modification: (1) no PVD; (2) partial PVD that includes VMA (Fig. 4D) and VMT (Fig. 4E); and (3) PVD. Vitreoschisis was defined when there was a split in the posterior vitreous cortex (Fig. 4F)^13^. HF were defined as discrete lesions protruding from the inner retinal surface (Fig. 4G). Epiretinal mass was defined as a homogenous collection of material with medium reflectivity at the inner retinal surface with a thickness greater than 20 µm (Fig. 4H)^10^. DMDRR was defined by the finding of a discrete margin of different retinal reflectivity in the mid- to far-peripheral retina on UWF-FP (Fig. 5)^11^.

### Statistical analysis

Student’s t-test was used for comparison of continuous variables between groups. Chi-square test was used for the comparison of categorical variables between groups. Cox proportional hazard ratios for the onset or development of PVD after cataract surgery in the presence of selected risk factors were calculated. SPSS version 15.0 for Windows (SPSS, Inc., Chicago, IL, USA) was used for statistical analysis. Descriptive data were recorded as means ± standard deviation unless otherwise specified. A *p*-value of < 0.05 was considered statistically significant.
